# An acoustic gap between the NICU and womb: a potential risk for compromised neuroplasticity of the auditory system in preterm infants

**DOI:** 10.3389/fnins.2014.00381

**Published:** 2014-12-05

**Authors:** Amir Lahav, Erika Skoe

**Affiliations:** ^1^Department of Pediatrics and Newborn Medicine, Brigham and Women's HospitalBoston, MA, USA; ^2^Department of Pediatrics, Harvard Medical School, MassGeneral Hospital for ChildrenBoston, MA, USA; ^3^Department of Speech, Language, and Hearing Sciences, Department of Psychology Affiliate, Cognitive Sciences Program Affiliate, University of ConnecticutStorrs, CT, USA

**Keywords:** auditory development, NICU, preterm infants, noise exposure, high frequency

## Abstract

The intrauterine environment allows the fetus to begin hearing low-frequency sounds in a protected fashion, ensuring initial optimal development of the peripheral and central auditory system. However, the auditory nursery provided by the womb vanishes once the preterm newborn enters the high-frequency (HF) noisy environment of the neonatal intensive care unit (NICU). The present article draws a concerning line between auditory system development and HF noise in the NICU, which we argue is not necessarily conducive to fostering this development. Overexposure to HF noise during critical periods disrupts the functional organization of auditory cortical circuits. As a result, we theorize that the ability to tune out noise and extract acoustic information in a noisy environment may be impaired, leading to increased risks for a variety of auditory, language, and attention disorders. Additionally, HF noise in the NICU often masks human speech sounds, further limiting quality exposure to linguistic stimuli. Understanding the impact of the sound environment on the developing auditory system is an important first step in meeting the developmental demands of preterm newborns undergoing intensive care.

## An acoustic gap between the NICU and the womb

Surrounded by amniotic fluid, the first sounds the fetus experiences are low-frequency digestive noises and maternal sounds transmitted through the bones of the skull (Querleu et al., 1988; Lecanuet and Schaal, 1996; Sohmer et al., 2001). However, preterm infants (born <37 weeks of gestation) are no longer surrounded by fluids or live underwater, and this new reality forces them to hear primarily through air conduction despite their auditory system being accustomed to bone conduction. This major difference in the primary mode of hearing (bone vs. air conduction) and the medium of sound transmission (fluid vs. air), presents an acoustic gap between the unnatural acoustic environment of the hospital and the developmental demands of the newborn's auditory system. The developmental implications of this acoustic gap remain largely unstudied. Differences between the auditory environments in the neonatal intensive care unit (NICU) vs. the womb are summarized in Table 1. Unlike the womb, the primary auditory stimulation available to intensive care neonates is environmental noise generated by ventilators, infusion pumps, fans, telephones, pagers, monitors, and alarms. Such excessive exposure to high-frequency noise, and recurrent electronic beeps that would not otherwise be present had the baby remained protected by the intrauterine environment and not been born prematurely, constitutes a trauma to the auditory system of a preterm infant. This acoustic trauma, we argue, may be potentially harmful, increasing the risk for auditory, language, and attention disorders. Although cases of hearing disorders in newborns are typically associated with congenital malformations, prenatal infections, and drug exposure (for review see Resendes et al., 2001; Beswick et al., 2012), this article is specifically focused on auditory impairments induced by environmental noise.

**Table 1:** **An acoustic gap between the NICU and the womb environments**.

	**Womb**	**NICU**
**Primary mode of hearing**	Bone conduction	Air conduction
**Sound transmission medium**	Fluid	Air
**Sound attenuation**	Attenuation provided by maternal tissue and fluids	Direct exposure to sound source
**Frequency range of sound exposure**	Primarily low frequency (<500 Hz)	Broad spectrum
**Ambient noise dosage**	Restricted daily exposure to noise	Excessive daily exposure to noise (e.g., alarms, white noise, and multi-talker babble)
**Most prevalent sounds in environment**	Maternal vocalizations, biological sounds (e.g., heartbeat, digestive noises)	Electronic, unnatural, non-biological sounds
**Exposure to language**	High-quality stimuli, primarily from mother	Poor quality stimuli during non-visiting hours, primarily from multi-talker babble
**Complexity of prevalent sounds in environment**	Rhythmic, periodic, organized, predictable (e.g., heartbeat)	Aperiodic (e.g., white noise), unorganized, unpredictable (e.g., alarms)

While exposure to loud noise is intuitively understood to be distracting and harmful, the shortage of biological and periodic auditory stimuli in the NICU environment is less acknowledged to be of concern. For example, the sensory perception of the maternal heartbeat in the womb provides the fetus with an important rhythmic experience that likely explains the natural tendency of the newborn to seek auditory entrainment soon after birth (Ingersoll and Thoman, 1994; Ullal-Gupta et al., 2013). In contrast, the more random, aperiodic nature of NICU noise suppresses opportunities for rhythmic entrainment known to facilitate arousal regulation (Smith and Steinschneider, 1975) and social interactions (Phillips-Silver et al., 2010) in early infancy.

## The frequency spectra in the NICU vs. the womb: implications for the tonotopic development of the auditory system

Auditory development is a slow process that begins *in utero*. Critical aspects of this development take place before full gestation and are therefore vulnerable to disruption by the NICU environment especially given that the frequency spectrum of the NICU environment is quite different from what is experienced in the womb. Previous studies have shown that the acoustic environment of the NICU contains a significant amount of HF noise (>500 Hz), emanating from a wide variety of medical equipment and human activity that are unlikely to be heard in the womb (Kellam and Bhatia, 2008; Livera et al., 2008). A recent study using sound spectral analysis over a five-day period showed that NICU infants were exposed to frequencies between 500 and 16,000 Hz 57% of the time, with the majority of exposure being during daytime falling in the range of 501–3150 Hz (Lahav, 2014). The potential risk of HF noise exposure in the NICU is further increased by the fact that the frequency spectra of NICU noise is rarely monitored, with majority of studies in the field solely focused on measuring loudness levels.

High-frequency frequency noise exposure in the NICU is a concern because the auditory system is still functionally underdeveloped at birth, with critical stages of development occurring during the final weeks of gestation (for review, see Graven and Browne, 2008). While the structural components of the inner ear (bony labyrinth of the cochlea) are already formed by 15 weeks gestational age (GA), the onset of cochlear function does not occur until 24 weeks GA or later (Pujol et al., 1991; Moore and Linthicum, 2007). As evidence of the functional onset of hearing, electrophysiological data from preterm neonates demonstrates that brainstem auditory evoked potentials are first recordable between 25 and 32 weeks GA (Starr et al., 1977; Amin et al., 2003; Yin et al., 2008; Coenraad et al., 2011; Jiang and Chen, 2014). After 34 weeks GA once the spiral ganglion neurons in the cochlea have formed sufficient neural connections with the auditory brainstem and have begun to extend those connections toward the auditory cortex, evoked potentials to sound become more robust (Pujol and Lavigne-Rebillard, 1992; Hepper and Shahidullah, 1994; Hall, 2000).

**Figure 1:**
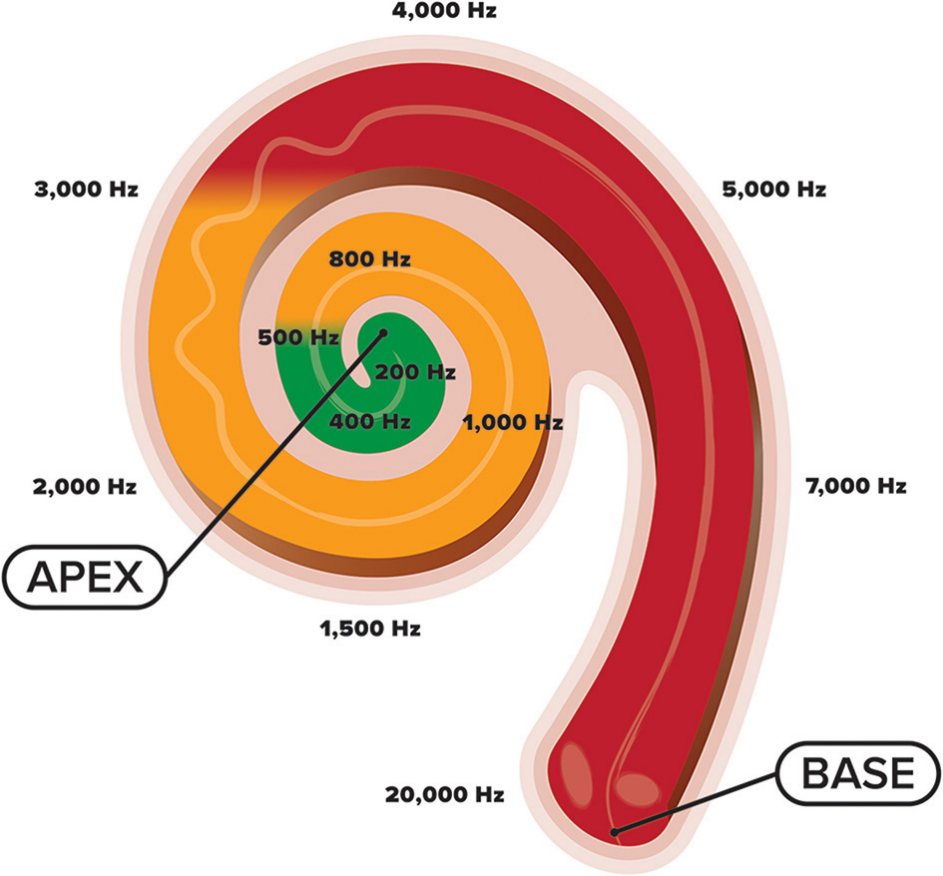
**An illustration of the cochlea and its tonotopic development across the frequency spectrum**. High-frequency sounds maximally stimulate the base of the cochlea, whereas low-frequency sounds maximally stimulate the apex. Whereas the fetus is primarily exposed to sound frequencies below 500 Hz (green shade), preterm newborns are exposed to the entire frequency spectrum (green, orange, and red shades), coming from various electronic sounds in the NICU environment.

Development of the cochlea and central auditory system is complex. Within the cochlea, reside tens of thousands of inner hair cells, sensory receptors, that each respond maximally to a specific frequency (Pujol et al., 1991; Pujol and Lavigne-Rebillard, 1992; Morlet et al., 1993). These hair cells are arranged tonotopically with high-frequency hair cells located basally (closer to the middle ear) and low-frequency ones located apically (Kandler et al., 2009) (see Figure 1). This cochlear tonotopy is preserved along the auditory neuroaxis as a consequence of spiral ganglion neurons establishing precise connections between cochlear hair cells and target neurons in the auditory brainstem that code for different sound frequencies (Pujol and Lavigne-Rebillard, 1992; Appler and Goodrich, 2011). Gradual development of these tonotopic frequency maps occurs with low-frequency regions maturing before high-frequency ones, a process often referred to as “frequency-dependent plasticity” (Talavage et al., 2000). This low-to-high developmental gradient is promoted by the acoustic makeup of the womb in which frequencies above 500 Hz are attenuated by maternal tissues and fluids within the intrauterine cavity. Toward the end of pregnancy, as the walls of the uterine lining begin to thin, gradually more HF energy (>500 Hz) is passed through the womb (Bench, 1968; Gerhardt, 1989; Gerhardt et al., 1990; Hepper and Shahidullah, 1994; Abrams and Gerhardt, 2000). Thus, while the womb provides an optimal medium for the initial phases of hearing development by limiting exposure to HF sounds (Hall, 2000), the sound frequencies present in the NICU are not necessarily conducive to furthering this development (Graven, 2000) (see Figure 1). Increased exposure to HF stimulation in the NICU while a majority of cochlear neurons are still migrating (Battin et al., 1998; Bystron et al., 2008) and cortical folding is still in flux, may disrupt the normal tonotopic tuning of cochlear hair cells, and hinder auditory development subcortically and cortically (Walker et al., 1971). Thus, owing to the experience-dependent nature of auditory development (Zhang et al., 2001; Chang and Merzenich, 2003; Oliver et al., 2011; Zhou et al., 2011), the statistical properties of the acoustic environment in the NICU may potentially misguide the topographic assembly of the auditory brain system (Pujol and Lavigne-Rebillard, 1992), resulting in poorer frequency resolution. It is therefore likely that overexposure to HF noise during this critical period may impede the developing auditory system with effects seen well-beyond the postnatal period.

## Can the fetus hear HF sounds originating outside of the womb?

As a consequence of the acoustic properties of the womb, the fetus receives, for the most part, a low-pass filtered version of the auditory environment in the world. Given the low-to-high frequency development of the cochlea, this raises the question of whether the fetus can in fact hear HF sounds that are loud enough to penetrate the womb and, if yes, when does this responsivity to HF sounds emerge? These questions have been addressed directly and indirectly by several studies using a variety of techniques. Hepper and Shahidullah (1994) examined the responsiveness of the human fetus to external auditory stimuli (pure tones) presented by a loudspeaker placed on the maternal abdomen at different frequencies (100, 250, 500, 1000, and 3000 Hz). Recording of fetal movements via ultrasound revealed a preferential sensitivity of the fetus to external sounds in the low-frequency range (<500 Hz) as early as 19 weeks of gestation. At 27 weeks GA, the vast majority of fetuses responded to sounds below 500 Hz but none responded to the higher frequency sounds at 1000 Hz or 3000 Hz. Responsiveness to sounds above 1000 Hz was not observed until 33 weeks gestation. For all frequencies presented, there was a significant decrease in the intensity required to elicit a response with increased GA, likely due to the maturation of the auditory system and the thinning of the intrauterine walls in the last trimester of the pregnancy (Querleu et al., 1988). A follow-up study by Kisilevsky et al. (2000) using high volume high-pass filtered white noise (800–20,000 Hz) presented to the mother's abdomen showed that sound-evoked responses (in the form of cardiac acceleration and body movement) emerged at 30 weeks for both low-risk and high-risk fetuses, and required less intense stimulation to evoke responses later in development. While both studies indicate that sensitivity to HF sound emerges during the 7–8th month of gestation, neither study examined auditory system function directly but instead used fetal movements as an indirect measurement of hearing sensitivity.

Studies using MEG- and fMRI-based techniques in fetuses provide more direct measurement of auditory cortical function to HF sounds presented at high intensity. This body of research has provided modest evidence that the auditory cortex is activated by frequencies above 500 Hz by 33 weeks (Draganova et al., 2005; Jardri et al., 2008), that by 33–36 weeks that the fetus can differentiate a 500 Hz sound from a higher frequency one (Draganova et al., 2005), and that later in gestation (37–41 weeks) the auditory cortex is activated by naturalistic sounds containing a broad spectrum of frequencies (Moore et al., 2001). Thus, the likelihood exists that by ~33 weeks, there is a degree of HF penetration of external sounds through the abdomen, allowing the fetal auditory system to be activated by the high-frequency aspects of speech and other complex, naturalistic stimulation. This prenatal exposure to HF naturalistic sounds has been argued to prime the fetus for voice recognition, vowel discrimination, melody discrimination, among other complex auditory skills (reviewed in Granier-Deferre et al., 2011).

Based on the studies reviewed in this section, it appears that fetuses can respond to HF sounds transmitted through the maternal abdomen after ~33 weeks, and that responsiveness increases with GA. However, the mere fact that fetuses are capable of responding to HF noise does not necessarily imply they should be exposed to such sounds, especially in high doses. The existing literature does not rule out the possibility that the intense exposure to HF sounds used in these specific experiments, especially during early stages of development, was in fact harmful. Therefore, the potential harm of intense, direct, and repeated exposure to HF noise without the protection of the maternal abdomen, as experienced by extremely preterm infants in the NICU, should be given a more carefully evaluation. However, unlike HF noise, exposure to potentially positive HF sounds (e.g., speech, music) during the last stages of gestation may in fact help set the stage for hearing and language skills.

## Noise-induced plasticity in the auditory system function

Our concern regarding the adverse effects of HF noise exposure is supported by evidence from animal studies. Animal models have revealed that sensory impoverishment during critical periods of development, in the form of acoustic noise or reduced complexity of auditory input, can lead to malformed tonotopic cortical maps, reduced neural synchrony, and broader tuning curves, which reflect decreased frequency-sensitivity of the auditory system (Zhang et al., 2001, 2002; Oliver et al., 2011). For example, young rats repeatedly exposed to HF tone pips showed distorted auditory function later in life (Oliver et al., 2011). The residual effects of that early augmented environment included altered brainstem auditory evoked potentials to the frequency of overstimulation, in addition to expanded neural frequency maps (Oliver et al., 2011). As a consequence of this early unnatural sound experience, the rat's auditory system became tuned to the frequency of the tone pips at the expense of processing other sound frequencies. Modification of tonotopic maps in deaf individuals (Guiraud et al., 2007) and musicians (Pantev et al., 1998), suggests that similar experience-dependent developmental principles operate in humans. It is therefore likely that the abrupt transition from the womb to the NICU changes the typical patterns of auditory development, specifically altering how frequency information is processed and coded.

In addition to compromising tonotopy, increased HF noise exposure during the neonatal period may have other long-term consequences for the functional integrity of the auditory system. In recent years, there has been growing concern about environmental noise in individuals of all ages. Indeed, sound intensities once thought to be safe for the auditory system are now considered less safe, especially for more extended exposures (Maison et al., 2013; Basner et al., 2014; Gourevitch et al., 2014). In laboratory animals, prolonged noise exposure has been shown to impede auditory development (Chang and Merzenich, 2003), accelerate age-related hearing losses (Kujawa and Liberman, 2006), increase neural loss (Salthouse and Lichty, 1985; Maison et al., 2013), and reduce neural efficiency by increasing the spontaneous firing of auditory neurons in the absence of sound stimulation (Costalupes et al., 1984; Seki and Eggermont, 2003). Moreover, in children, excessive noise exposure can manifest in decreased reading and cognitive performance (Cohen et al., 1973; Bronzaft and McCarthy, 1975; Hygge et al., 2003), and may change how children discriminate and attend to auditory stimuli (Cohen et al., 1973; Evans and Kantrowitz, 2002; Evans et al., 2009), even when tested in quiet environments.

Considering the acoustic gap between the NICU environment and the womb, it is not surprising that auditory development is compromised in preterm compared to full-term newborns. Studies using brainstem auditory evoked potentials suggest that preterm infants have delayed myelination of the central auditory pathway (Pasman et al., 1996; Roopakala et al., 2011; Hasani and Jafari, 2013) in addition to atypical neural pathways when processing, discriminating, and memorizing auditory information (Fellman et al., 2004; Therien et al., 2004). Thus, exposing preterm infants to HF noise too early, while the auditory system is still immature, may hinder the normal development of hearing and subsequent language acquisition.

## Increased behavioral relevance of HF noise as a consequence of NICU experience

In addition to the presumably harmful effects of HF noise exposure to auditory system development, the collection of electronic noises in the NICU environment (coming from ventilators, telephones, pagers, and alarms) can often produce sufficient acoustic energy to mask natural human speech sounds potentially important to the preterm infant, whose exposure to linguistic stimuli is already restricted. This impoverished linguistic experience increases the behavioral relevance of noise, by shifting attentional focus away from speech sounds toward the noise in the environment. Behavioral and neurophysiological data from fetuses and healthy newborns, have revealed that fetuses become sensitive to sounds in the environment that are transmitted through the amniotic fluid including the sound of the mother's and father's voices (Fifer and Moon, 1994; Kisilevsky et al., 2003; Beauchemin et al., 2011; Voegtline et al., 2013; Lee and Kisilevsky, 2014) (reviewed in, Fava et al., 2011), with evidence of experience-dependent auditory learning emerging before birth (Kujala et al., 2003; Partanen et al., 2013; Krueger and Garvan, 2014). Given the importance of early experience in molding the auditory system (Skoe and Chandrasekaran, 2014), increased exposure to noise may over sensitize infants to noise, and, as a consequence, neural circuits may be formed to make noise the primary target of attention rather than treating it as a background stimulus that should be ignored. While this is an intriguing possibility, further research is needed to confirm or dispute this hypothesis.

Another factor that may impede the preterm infant's ability to tune out noise, is the immaturity of auditory feedback mechanisms (Morlet et al., 1993; Graven and Browne, 2008). In addition to inner hair cells, the cochlea contains outer hair cells that receive feedback from the central auditory system that buffer noise-induced damage and improve speech intelligibility in noise (Guinan, 2006). Background noise leads to a decrease in speech intelligibility that poses a perceptual challenge even for healthy adults with normal hearing. In addition to masking the signal due to physical overlap between the acoustics of noise and the acoustics of speech, noise acts as a competing signal that interferes with the ability to attend to a concurrent speech stream (Assmann and Summerfield, 1999). If greater behavioral relevance (i.e., unconscious attention) is placed on noise in the environment, or if biological feedback mechanisms are not fully intact, this could create further challenges for processing speech in noise for the preterm infant both in the immediate and also later in life. In support of this possibility, studies using brainstem auditory evoked potentials suggest that preterm infants have delayed myelination of the central auditory pathways (Pasman et al., 1996; Roopakala et al., 2011), which is associated with a variety auditory processing disorders (APD). Whether or not the high prevalence of APD in preterm population is attributed to the presence of high-frequency noise in the NICU is undetermined. However, one of the hallmarks of APD is difficulty processing target signals within a background of noise (Keith, 1999), further supporting the possibility that exposure to HF noise of the NICU environment may impede the preterm infant's ability to pull out signals from noise.

## Optimal frequency exposure for intensive care neonates: lack of recommended standards

Current guidelines set by the American Academy of Pediatrics (AAP) are primarily focused on loudness levels, leaving the potential risks of HF noise exposure in the NICU infants largely unaddressed. According to AAP standards (White et al., 2013), the combination of continuous background sound and operational sound shall not exceed an hourly Leq of 45 dB and an hourly L10 of 50 dB, while transient sounds (Lmax) shall not exceed 65 dB, all A-weighted slow response measurements (White et al., 2013). However, in practice, previous studies examining noise in the NICU have reported extremely high noise levels, exceeding the AAP recommended standards more than 70% of the time (Williams et al., 2007). Sound measurements within the NICU environment have been measured between 62 and 70 dBA (Philbin and Gray, 2002), with peak impulses exceeding 90 dBA (Williams et al., 2007) and 120 dBA (Kent et al., 2002). In another study, sound measurements yielded an overall average hourly level (Leq) of approximately 60 dBA with peak levels (Lmax) of 78.39 dBA (Krueger et al., 2005).

The problem of loud noise in the NICU has been diminished by modifications to NICU architectural designs, as more hospitals transition toward the single-room model in which newborns are housed in private rooms vs. the open-bay model where multiple babies are co-cared in a large room (White, 2011). Studies have shown that private-room NICUs are generally quieter than open-bay NICUs (Szymczak and Shellhaas, 2014), except when high–frequency ventilation is used (Liu, 2012). However, while private rooms may have more favorable acoustics than open-bay designs, care should still be taken to ensure that the peak intensity and frequency characteristics in the NICU environment, even within a private room, are still optimal for preterm newborns. Thus, in the absence of clear guidelines and recommendations regarding the acoustic makeup of optimal sound exposure at birth, the NICU may present an acoustic danger zone for preterm newborns.

## Lifelong auditory plasticity: recovery options for preterm infants

While experience-dependent plasticity is greatest in the early years, the auditory system maintains the potential for malleability throughout life (Sanes and Woolley, 2011). For example, auditory brain plasticity has been demonstrated in older adults following short-term sound-based training (Tremblay et al., 2001; Song et al., 2008; Anderson et al., 2013). Similarly, musical training has been shown improve linguistic and cognitive abilities (Moreno et al., 2009; Strait et al., 2014) and speech intelligibility in noise (Strait et al., 2012) in young children, leading to neural enhancements of brain structure and function (Hyde et al., 2009; Halwani et al., 2011; Ellis et al., 2012; Strait and Kraus, 2014), and buffering against auditory aging in older adults (Parbery-Clark et al., 2009, 2012). In addition, cochlear implants can induce functional plasticity in the auditory brainstem even after many years of deafness in childhood, demonstrating the high degree of modifiability in brain mechanisms that support hearing abilities (Gordon et al., 2011; Cardon et al., 2012). Thus, our auditory histories—whether in the form of excessive noise, acoustic deprivation, or augmented sound training—can influence auditory processes across the lifespan (Skoe and Chandrasekaran, 2014).

What are the implications of this lifelong plasticity for preterm infants following NICU discharge? While the NICU environment may initially compromise the auditory development, it is encouraging that the post-NICU environment may help to close the developmental gap by allowing for near normal to normal auditory functionality later in life. Enriched home literacy environment and quality exposure to auditory and linguistic stimuli in the post-NICU environment are considered fundamental building blocks for this auditory neuroplasticity, laying the foundation for speech and language development (Burgess et al., 2002; Roberts et al., 2005; Rowe and Goldin-Meadow, 2009; Hart and Risley, 2010; Hammer et al., 2010; Skoe et al., 2013; Ramirez-Esparza et al., 2014). Although hearing, language, and attention deficits are common among preterm infants (Vohr, 2014), the fact that some children born prematurely manage to catch up to their peers suggests that despite the initial auditory trauma induced by the NICU environment, the window of opportunities for further plasticity and recovery remains open.

## Conclusions

The acoustic gap between the NICU and the womb, although somewhat unescapable, poses a hazard that may disrupt auditory development in intensive care neonates. As a consequence of the NICU environment, preterm infants receive a heavier dose of HF noise than what would be normally possible in the womb. The long-term effects of HF noise exposure on the development of preterm newborns prior to full gestation development is a growing area of research of particular clinical importance. The negative plasticity of the auditory brain system in response to HF noise exposure is concerning and highlights the importance of the newborn's sensory experience during postnatal hospitalization. It is tempting to theorize that excessive exposure to high-frequency noise during critical periods may be a contributing factor to the language, attention, and cognitive deficits often seen in the preterm population. Despite these evident concerns regarding HF noise exposure, current guidelines set by the AAP (White et al., 2013) are primarily focused on loudness levels, leaving the potential risks of HF noise exposure in the NICU largely overlooked. More knowledge of the spectral content of NICU noise would help in evaluating the auditory developmental consequences in NICU graduates. Intensive care neonates deserve to have a better protection plan against toxic sounds.

### Conflict of interest statement

The authors declare that the research was conducted in the absence of any commercial or financial relationships that could be construed as a potential conflict of interest.
